# Prediction of pulmonary tuberculosis case trends among older adults in Chongqing based on time series models

**DOI:** 10.3389/fpubh.2026.1839570

**Published:** 2026-05-18

**Authors:** Bojie Gao, Shanrong Huang, Jie Luo, Wenping Liao, Yu Xin, Juan Lv, Lin Hu, Ping Zhang, Wen Zhang, Chuan Pu

**Affiliations:** 1 School of Public Health, Chongqing Medical University, Chongqing, China; 2 Chongqing Institute of Tuberculosis Control and Prevention, Chongqing, China

**Keywords:** older adults, hybrid model, NNAR, pulmonary tuberculosis, SARIMA

## Abstract

**Background:**

Tuberculosis is a major global public health issue. Older adult individuals, due to factors like immunosenescence and comorbidities, are at high risk for TB. Chongqing’s significant aging population poses severe challenges for TB control in this group.

**Objective:**

This study is based on the monthly case counts of pulmonary tuberculosis among older adults aged 65 and above in Chongqing from January 2020 to June 2024. It constructs and compares the Seasonal Autoregressive Integrated Moving Average (SARIMA) model, the Nonlinear Autoregressive Neural Network (NNAR) model, and the hybrid SARIMA-NNAR model to predict the monthly number of PTB cases in 2025.

**Methods:**

The study data were extracted from the National Tuberculosis Surveillance System (TBIMS). Data collection and organization for pulmonary tuberculosis cases among individuals aged 65 and above were performed using Microsoft Excel 2019 (Microsoft Corp). Statistical analysis and predictive modeling were conducted using R software, version 4.5.2 (Network Theory Ltd., Bristol, United Kingdom). Data from January 2020 to June 2024 formed the training set, while July to December 2024 data served as the testing set. Model performance was evaluated using Root Mean Square Error (RMSE), Mean Absolute Error (MAE), and Mean Absolute Percentage Error (MAPE).

**Results:**

The number of pulmonary tuberculosis cases among older adults in Chongqing exhibited distinct seasonal fluctuations, with peaks consistently occurring in March and May each year. On the testing set, the hybrid model achieved the lowest MAE (38.39%) and MAPE (11.77%), whereas the NNAR model produced the lowest RMSE (45.52%). Overall, the hybrid model demonstrated a more balanced performance across evaluation metrics. The forecasted case counts for 2025 maintained a similar seasonal pattern, with projected peaks in March and May.

**Conclusion:**

The SARIMA-NNAR hybrid model improves the prediction accuracy of pulmonary tuberculosis case counts in older adults by integrating linear and nonlinear components, providing a scientific basis for optimizing resource allocation and seasonal interventions in Chongqing.

## 1 Introduction

Tuberculosis (TB), caused by Mycobacterium TB, is a chronic infectious disease that poses a serious threat to public health and represents a major global public health challenge (1). According to the World Health Organization (WHO) report (1), an estimated 10.7 million people fell ill with TB globally in 2024. Despite significant progress in global control efforts, TB remains one of the leading infectious causes of morbidity and mortality worldwide, particularly among older adults. The report indicates that in 2024, approximately 1.43 million TB cases occurred in individuals aged 65 and above, with about 0.5 million among women and 0.93 million among men. The Western Pacific Region recorded the highest number of cases in this age group. Older adults, characterized by declining physiological functions, immunosenescence, and a high prevalence of comorbid chronic underlying diseases, constitute a high-risk population for TB infection, disease progression, and recurrence. This demographic poses a severe challenge to TB epidemic control (2–4).

As a high TB burden country, China had approximately 696,000 TB cases in 2024, ranking fourth globally. The number of patients in the population aged 65 and above was approximately 245,000 (1). Chongqing, as a municipality in southwestern China, has a large population base and a severe degree of aging. By 2024, the population aged 65 and above in Chongqing reached 6.0204 million, accounting for 18.87% of the total population of Chongqing (5). In recent years, Chongqing has invested substantial resources in TB prevention and control, and the overall incidence has shown a downward trend (6). However, the unique geographical environment of Chongqing further exacerbates the prevention and control of TB among older adult population, particularly pulmonary tuberculosis (PTB) in older adults. Issues such as uneven distribution of medical resources and poor accessibility of medications in remote areas increase the difficulty of prevention and control (7, 8). A recent nationwide study further reported that, after removing the influence of COVID-19, the western region of China, where Chongqing is located, continues to face a disproportionately high TB burden and is unlikely to meet the WHO 2025 targets (9), underscoring the urgency of strengthening region-specific forecasting for high-risk populations such as older adults. Therefore, conducting systematic analysis and trend prediction of the case patterns of PTB among older adults in Chongqing can provide certain reference value for optimizing regional prevention and control strategies and reducing the disease burden.

ARIMA and SARIMA models are traditional and classical forecasting models. Based on linear assumptions, they are used to study data that changes over time (10). These models capture the dynamic patterns of diseases by extracting trends, seasonality, and periodicity from historical disease data, thereby establishing suitable mathematical models (11). Among them, the traditional Autoregressive Integrated Moving Average (ARIMA) model has been widely applied, but its modeling requires the time series to exhibit linear characteristics. However, real-world time series often possess uncertainty and complexity, especially in epidemiological time series, which frequently contain both linear and nonlinear structures (12–14). Although the Seasonal Autoregressive Integrated Moving Average (SARIMA) model can handle seasonal variations, its ability to fit non-stationary time series remains limited (15). In TB forecasting, SARIMA has been applied at the national level in China and shown to achieve strong predictive performance (9).

Artificial Neural Networks (ANNs) possess adaptive and nonlinear characteristics, making them suitable for uncovering nonlinear relationships within time series data. Their nonlinear mapping capabilities provide high predictive accuracy (16, 17). The Nonlinear Autoregressive Neural Network (NARNN), known for its strong fault tolerance, is widely used for time series forecasting and is also referred to as the Neural Network Autoregression (NNAR) model (18). However, some studies have indicated that ANNs may underperform traditional linear models when handling purely linear patterns. Therefore, relying solely on ANNs may not be the optimal approach for solving practical time series forecasting problems (19, 20).

In recent years, to overcome the limitations of individual models and enhance prediction accuracy, hybrid models have gained increasing attention. A common approach is to combine SARIMA with NNAR, allowing SARIMA to capture the linear components within the sequence while NNAR fits the nonlinear residuals, thereby leveraging the strengths of each model (21, 22). Such hybrid models have been applied in PTB forecasting and have demonstrated higher predictive accuracy (23). However, some researchers have also noted that the performance of hybrid models does not always surpass that of their individual components, as the effectiveness of model integration depends on specific data characteristics and modeling methodologies (24). The recognition that hybrid strategies hold promise but require careful evaluation extends well beyond epidemiological forecasting.

From a methodological perspective, the integration of linear statistical models with neural network components has gained traction across diverse scientific disciplines. Sharma et al. demonstrated that artificial neural network architectures can accurately capture nonlinear dynamics in complex systems (25). Subsequent studies have shown that AI-driven corrections can substantially enhance the predictive performance of baseline models (26), that systematic multi-model comparison is essential for identifying optimal architectures (27), and that machine learning methods can robustly model nonlinear patterns even when the underlying processes are incompletely understood (28, 29). These cross-disciplinary advances illustrate a common methodological principle: combining a structured baseline model with a flexible data-driven component can improve predictive accuracy beyond what either approach achieves alone.

Therefore, based on surveillance data from older adult population aged 65 and above in Chongqing from 2020 to 2024, this study aims to conduct a dedicated trend analysis of the epidemiological characteristics of PTB in this demographic. It will construct SARIMA, NNAR, and SARIMA-NNAR hybrid models for short-term forecasting. By comparing the predictive performance of different models, the study seeks to explore the epidemic patterns of PTB among older adults, thereby providing a scientific basis for formulating more targeted prevention and control strategies.

## 2 Materials and methods

### 2.1 Data collection

The data for this study were obtained from the China Tuberculosis Information Management System (TBIMS). This system collected reported data on newly diagnosed pulmonary tuberculosis(PTB) patients aged 65 and above from healthcare institutions across 38 districts and counties in Chongqing Municipality, covering the period from January 1, 2020, to December 31, 2024. To protect patient privacy and prevent duplicate reporting, each reported TB case was assigned a unique ID. Population data for individuals aged 65 and above in each district and county of Chongqing from 2020 to 2024 were sourced from the Chongqing Statistical Yearbook (30) and the Chongqing Statistical Bulletin on National Economic and Social Development (5). The monthly number of PTB cases among older adults from January 2020 to June 2024 constituted the training set for model fitting. Data from July to December 2024 formed the testing set, which was used to evaluate model performance. The predictive performance of the models was assessed using the following metrics: Root Mean Square Error (RMSE), Mean Absolute Error (MAE), and Mean Absolute Percentage Error (MAPE). The constructed models were subsequently employed to forecast the number of older adults PTB cases from January to December 2025.

### 2.2 SARIMA model

The SARIMA model is a classical statistical method widely used for forecasting time series data with trends and seasonal variations. By explicitly incorporating seasonal components, it extends the ARIMA model and is therefore suitable for disease case time series data that often exhibit regular periodic fluctuations. The SARIMA model is denoted as SARIMA(p, d, q)(P, D, Q)[S], where p = order of the non-seasonal autoregressive (AR) component, d = degree of non-seasonal differencing, q = order of the non-seasonal moving average (MA) component, P = order of the seasonal autoregressive (AR) component, D = degree of seasonal differencing, Q = order of the seasonal moving average (MA) component, and S = length of the seasonal period (in this study, monthly data are used, S = 12). The expression of the SARIMA model is shown in Equation 1 (31):

$$\Phi (B^{S})\phi (B){\left(1-B^{S}\right)}^{D}{\left(1-B\right)}^{d}y_{t}=\Theta (B^{S})\theta (B)\varepsilon_{t}$$ (1)

The nonseasonal components are shown in Equation 2:

$$\text{AR:}\phi (B)=1-\phi_{1}B-\cdots -\phi_{p}B^{p}$$

$$\text{MR:}\;\theta (B)=1-\theta_{1}B-\cdots -\theta_{q}B^{q}\;$$ (2)

The seasonal components are shown in Equation 3:

$$\text{Seasonal}\;\mathrm{AR}:\Phi (B^{S})=1-\Phi_{1}B^{S}-\cdots -\Phi_{P}B^{\mathrm{PS}.}$$

$$\text{Seasonal}\;\mathrm{MR}:\Theta (B^{S})=1-\Theta_{1}B^{S}-\cdots -\Theta_{Q}B^{\mathrm{QS}}$$ (3)

$y_{t}\;$ is the observed value at time t, *B* denotes the backshift operator, and $\varepsilon_{t}$ is the residual at time t.

SARIMA modeling follows a standard procedure to ensure the robustness of the model (32, 33): First, the Augmented Dickey–Fuller (ADF) unit root test is performed on the original series to assess stationarity; non-stationary series are transformed into stationary series through regular differencing and seasonal differencing. Next, the autocorrelation function (ACF) and partial autocorrelation function (PACF) are used to preliminarily identify the order of the SARIMA model based on truncation or trailing characteristics. The optimal model is selected by minimizing the Akaike Information Criterion (AIC) and the Bayesian Information Criterion (BIC), followed by parameter estimation. Finally, the residuals of the fitted model are examined using ACF, PACF, and the Ljung–Box test to ensure that the residuals are white noise and exhibit no autocorrelation.

The automatic order selection for this time series was performed using the “auto.arima” function from the “forecast” package in R, based on the Akaike Information Criterion (AIC). The fitted model obtained when the AIC value is minimized was selected as the optimal fitting model.

The automatic order selection for this time series was performed using the auto.arima() function from the forecast package in R. The function was executed with the arguments seasonal = TRUE, stepwise = FALSE, approximation = FALSE, and trace = FALSE to ensure an exhaustive search over all candidate models. Model selection was based on minimization of the Akaike Information Criterion (AIC). Model adequacy was assessed using the Ljung–Box test with lag = 12 to examine residual autocorrelation over one full seasonal cycle.

### 2.3 NNAR model

Artificial Neural Networks are mathematical models that simulate biological neural networks, serving as a nonlinear time series forecasting method based on feedforward neural networks and widely applied to complex nonlinear predictions. Their basic structure consists of an input layer (input variables), an output layer (output variables), and hidden layers (layers of nodes between the input and output layers). The constructed neural network is referred to as an NNAR(p, P, k) model, where p represents the non-seasonal lag order, P denotes the seasonal lag order, and k is the number of hidden nodes. For seasonal time series, it can be expressed as NNAR(p, P, k)m, with m being the length of the seasonal period. Through the nonlinear transformation of the hidden layers, this structure enables the model to capture complex dynamic patterns within the time series, thereby overcoming the limitations of traditional linear autoregressive models. The expression of the NNAR model is shown in Equation 4 (31):

$$y_{t}=f(y_{t-1},y_{t-2},\ldots ,y_{t-p},y_{t-m},y_{t-2m},\ldots ,y_{t-\mathrm{Pm}})+\varepsilon_{t}$$ (4)

$y_{t}$ is the observed value at time t, and $\varepsilon_{t}$ is the residual at time t.

**Figure 1:**
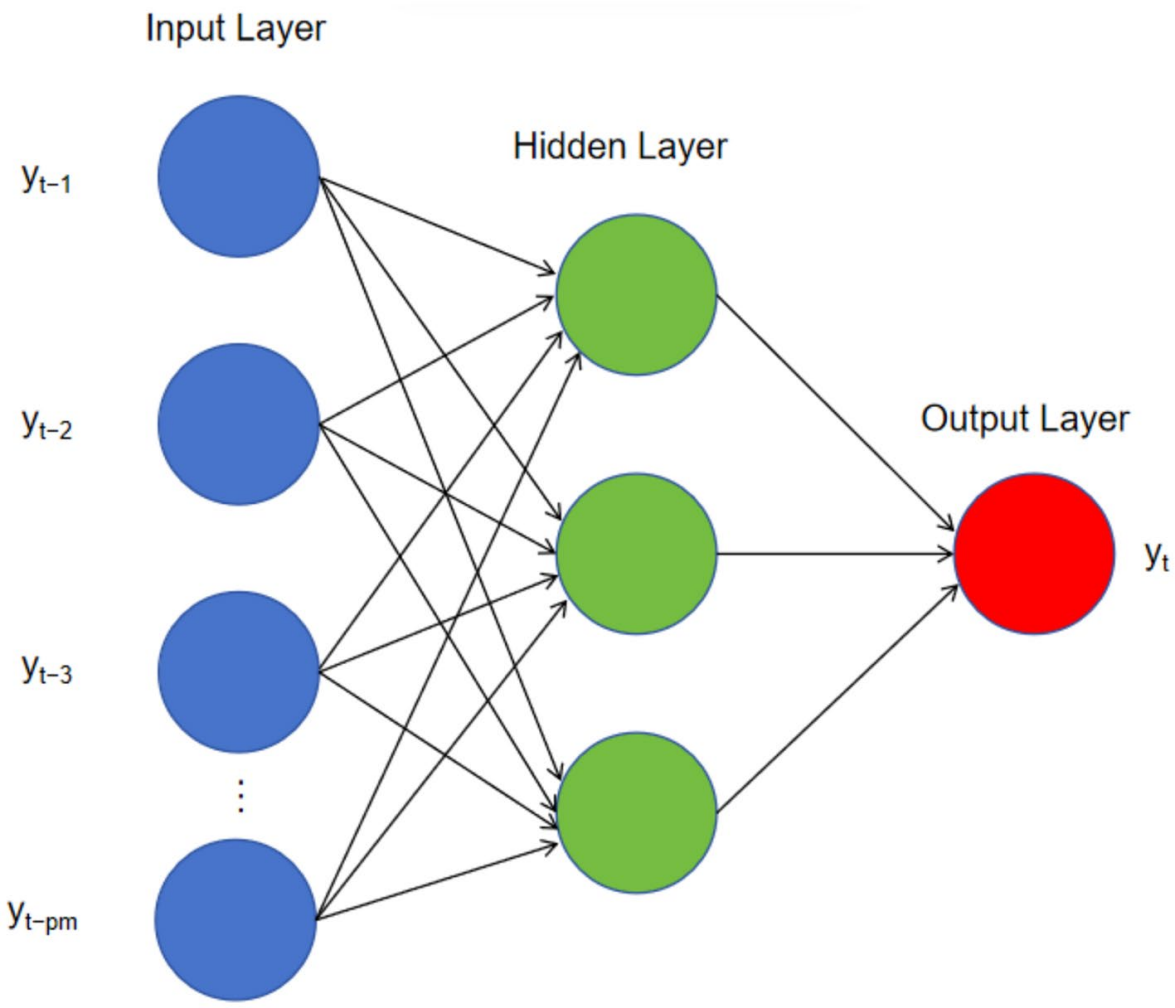
NNAR neural network diagram.

For seasonal time series, the nnetar() function from the forecast package automatically sets the seasonal lag order to *p* = 1. The non-seasonal lag order p is selected based on the optimal linear model fitted to the seasonally adjusted series. The number of hidden nodes k was determined by the function’s default rule: k = (p + P + 1)/2. To ensure reproducibility, a random seed was fixed with set.seed(123) prior to model training. Prediction intervals (80 and 95%) were generated via bootstrap resampling of the residuals, as implemented in the forecast.nnetar() function(Figure 1).

### 2.4 Hybrid (SARIMA-NNAR) model

**Figure 2:**
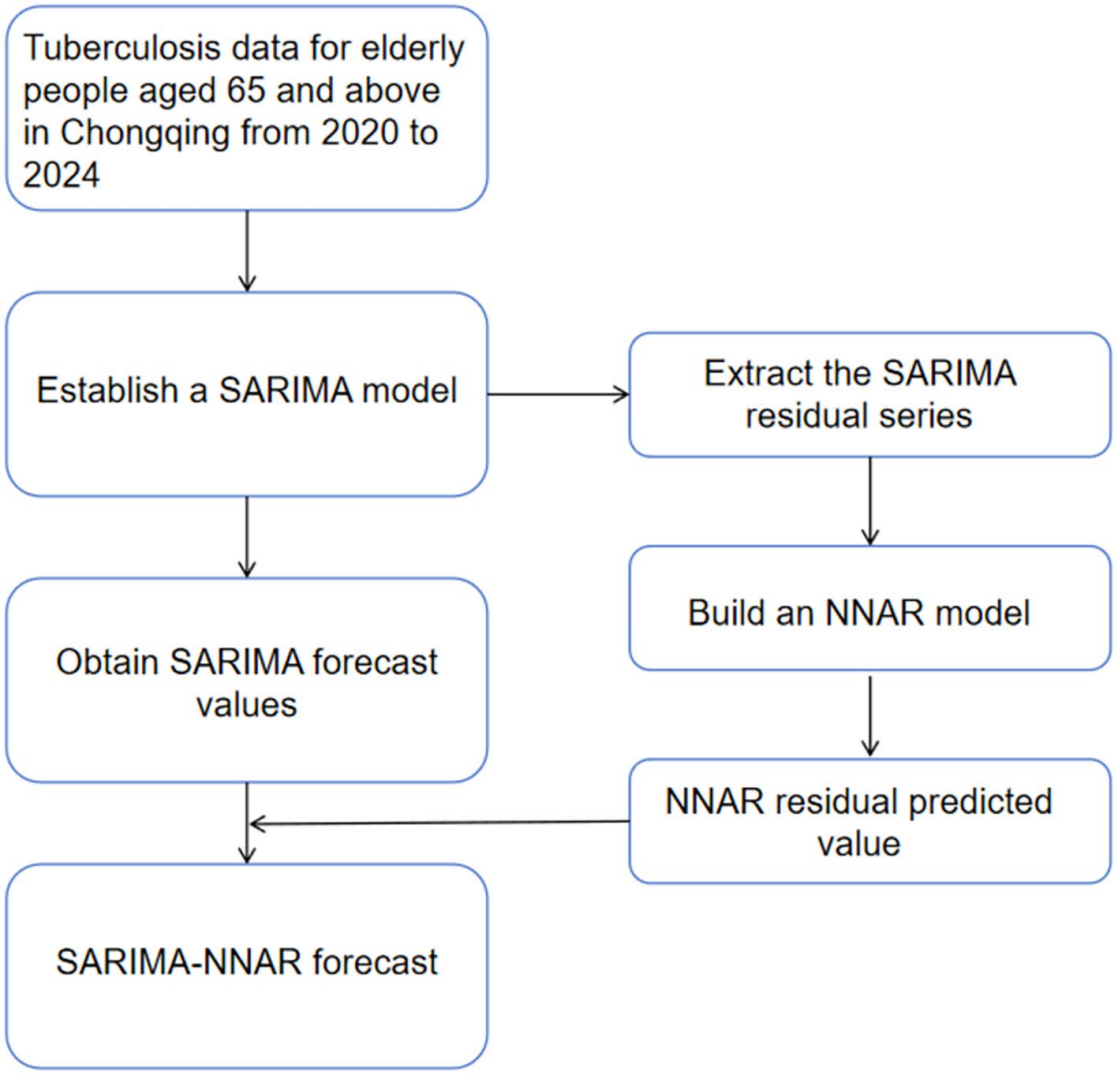
SARIMA-NNAR combined model flowchart.

Hybrid models incorporate both linear and nonlinear autocorrelation components. The SARIMA and NNAR methods work in synergy, utilizing observed past time series data to predict future values, making them suitable for addressing both nonlinear and linear problems. This study employs a hybrid model that combines the SARIMA model (linear) and NNAR (nonlinear). First, a SARIMA model was fitted to the entire training series, and the in-sample residuals were extracted. These residuals were treated as a new univariate time series and tested for stationarity using the Augmented Dickey–Fuller test. Second, an NNAR model with the same structure as the standalone NNAR model was fitted to the residual series using the same random seed (set.seed(123)). The NNAR component was trained solely on the lagged values of these residuals, without including lagged terms of the original case series, ensuring that the nonlinear modeling was confined to the residual structure not captured by SARIMA. Both models were fitted once and then held fixed; no iterative re-fitting or updating was performed between the two stages or during forecasting. For any future time point, the final hybrid point forecast was computed as the sum of the SARIMA forecast and the NNAR residual forecast (34). Prediction intervals for the hybrid model were obtained by adding the point-wise intervals from the SARIMA and NNAR forecasts, under the assumption of independence between the linear and nonlinear error components(Figure 2).

### 2.5 Measures of accuracy

For three different models, selecting appropriate evaluation metrics can better measure model performance and estimate prediction accuracy. This study uses Root Mean Square Error (RMSE), Mean Absolute Error (MAE), and Mean Absolute Percentage Error (MAPE) as model selection criteria as defined in Equations 5‒7. The expressions for the above metrics are, respectively, shown below (23):

$$\text{RMSE}=\sqrt{\frac{1}{n}\sum_{t=1}^{n}{\left(y_{t}-{\hat{y}}_{t}\right)}^{2}}$$ (5)

$$\mathrm{MAE}=\frac{1}{n}\sum_{t=1}^{n}|y_{t}-{\hat{y}}_{t}|$$ (6)

$$\text{MAPE}=\frac{1}{n}\sum_{t=1}^{n}|\frac{y_{t}-{\hat{y}}_{t}}{y_{t}}|\times 100\%$$ (7)

### 2.6 Data processing and analysis

Data collection and organization for PTB cases among individuals aged 65 and above were performed using Microsoft Excel 2019 (Microsoft Corp). Statistical analysis and predictive modeling were conducted using R software, version 4.5.2 (Network Theory Ltd., Bristol, United Kingdom). A *p* < 0.05 was considered statistically significant.

## 3 Results

A total of 19,607 cases of PTB in individuals aged 65 and above were registered in Chongqing from 2020 to 2024. The annual case counts were 3,982, 3,994, 3,659, 3,974, and 3,998, respectively, with an average of 3,921 cases per year. The average annual incidence rate was 67.34 per 100,000 population (Table 1). From 2020 to 2024, the number of TB cases exhibited seasonal variation. The number of pulmonary tuberculosis cases among older adults in Chongqing exhibited distinct seasonal fluctuations, with peaks consistently observed in March and May.

**Table 1:** Monthly reported cases of PTB from January 2020 to December 2024.

Months	2020	2021	2022	2023	2024
January	267	279	279	283	337
February	164	286	267	356	291
March	379	381	363	391	378
April	376	347	331	406	346
May	382	379	335	375	372
June	395	402	313	350	317
July	436	370	370	357	370
August	334	335	397	317	314
September	353	359	349	327	323
October	274	262	289	303	291
November	302	285	188	274	324
December	320	309	158	235	335

### 3.1 Performance of the SARIMA model

**Figure 3:**
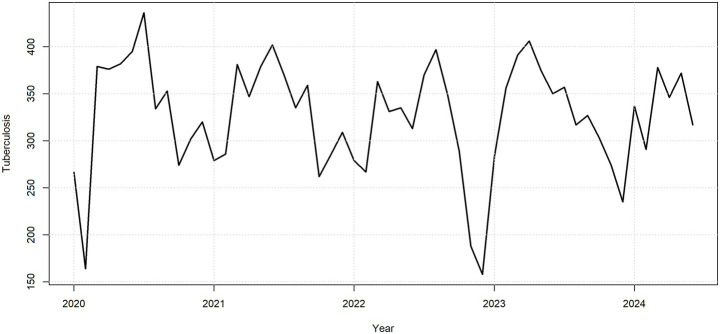
Time series of monthly reported PTB cases in the training set (January 2020–June 2024).

**Figure 4:**
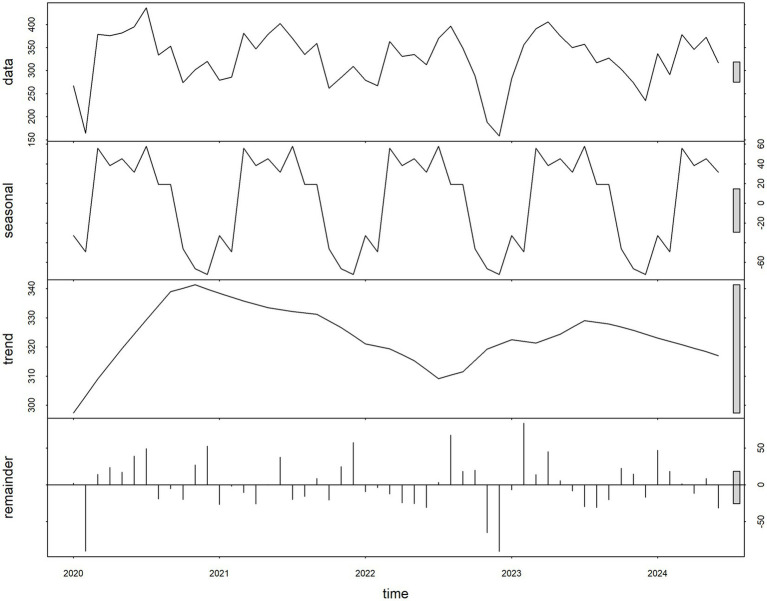
Additive decomposition of a time series graph.

**Figure 5:**
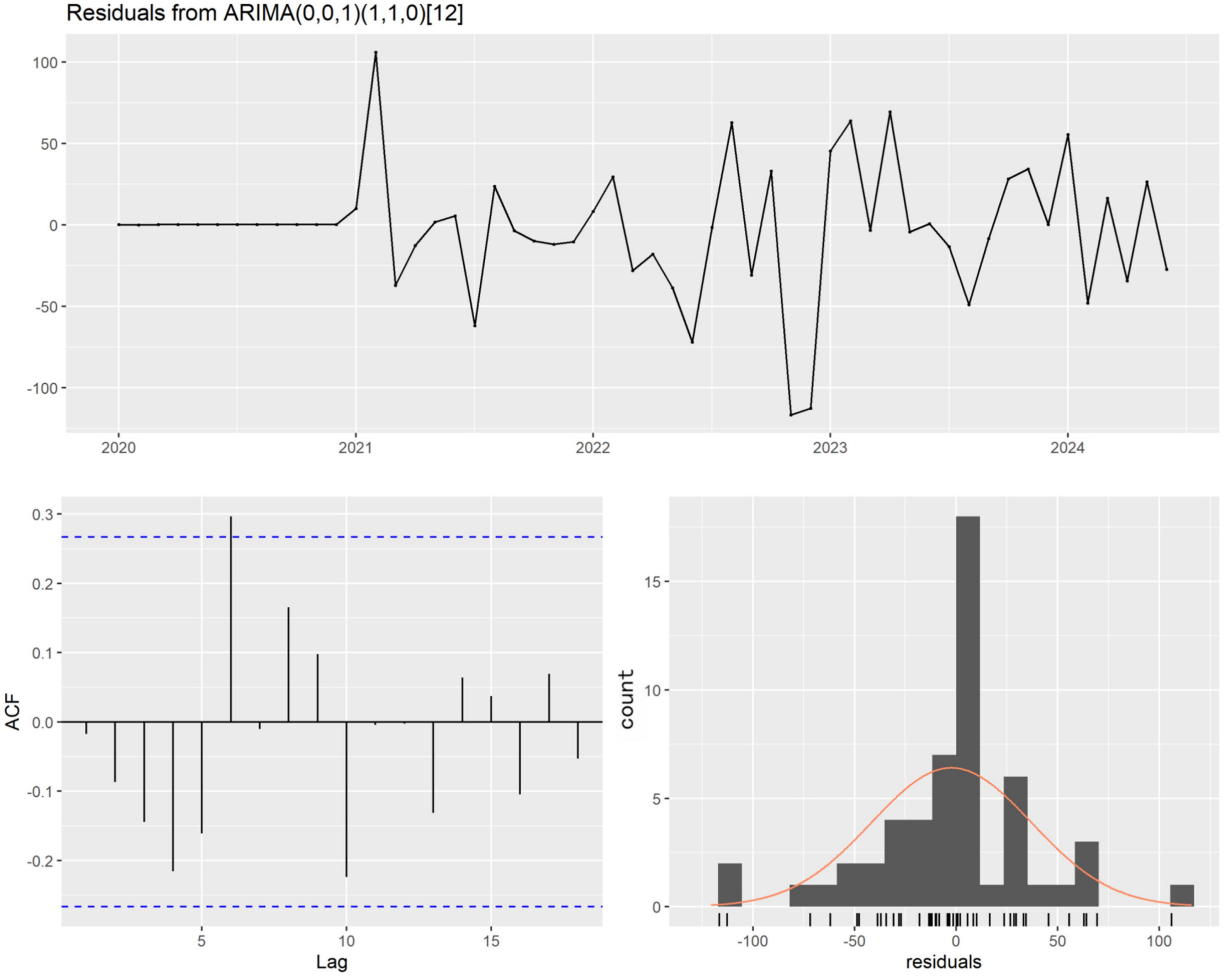
Residual plot, corresponding ACF plot, and histogram of SARIMA (0,0,1)(1,1,0)_12_.

This study focused on the comparative analysis and forecasting of time series data of PTB case counts. Prior to model fitting, a time series plot was drawn (Figure 3) to assess the data distribution over the five-year period, and the Augmented Dickey-Fuller (ADF) test was performed on the training set. The results indicated that the time series was stationary (*p* = 0.01). Additive decomposition was applied to the PTB time series to describe its seasonal components and trend. The results revealed that the PTB case count data exhibited seasonal variation (Figure 4). The most appropriate model was selected using the auto.arima() function. The optimal model obtained was SARIMA (0,0,1)(1,1,0) (12), with the lowest AIC of 446.23 and a BIC of 451.44. Although the ADF test confirmed non-seasonal stationarity, seasonal differencing (D = 1) was independently selected by the AIC criterion to adequately capture the seasonal structure of the series. The Ljung–Box test was conducted on this model, yielding a *p*-value of 0.0631, indicating no autocorrelation in the residuals (Figure 5). The residual ACF plot and PACF plot (Supplementary Figure S1) further confirmed that all autocorrelation and partial autocorrelation coefficients at lags 1–24 fell within the 95% confidence bounds, demonstrating that the SARIMA component successfully extracted all systematic linear and seasonal structure from the data before the NNAR residual modeling stage.

### 3.2 Performance of the NNAR model

**Figure 6:**
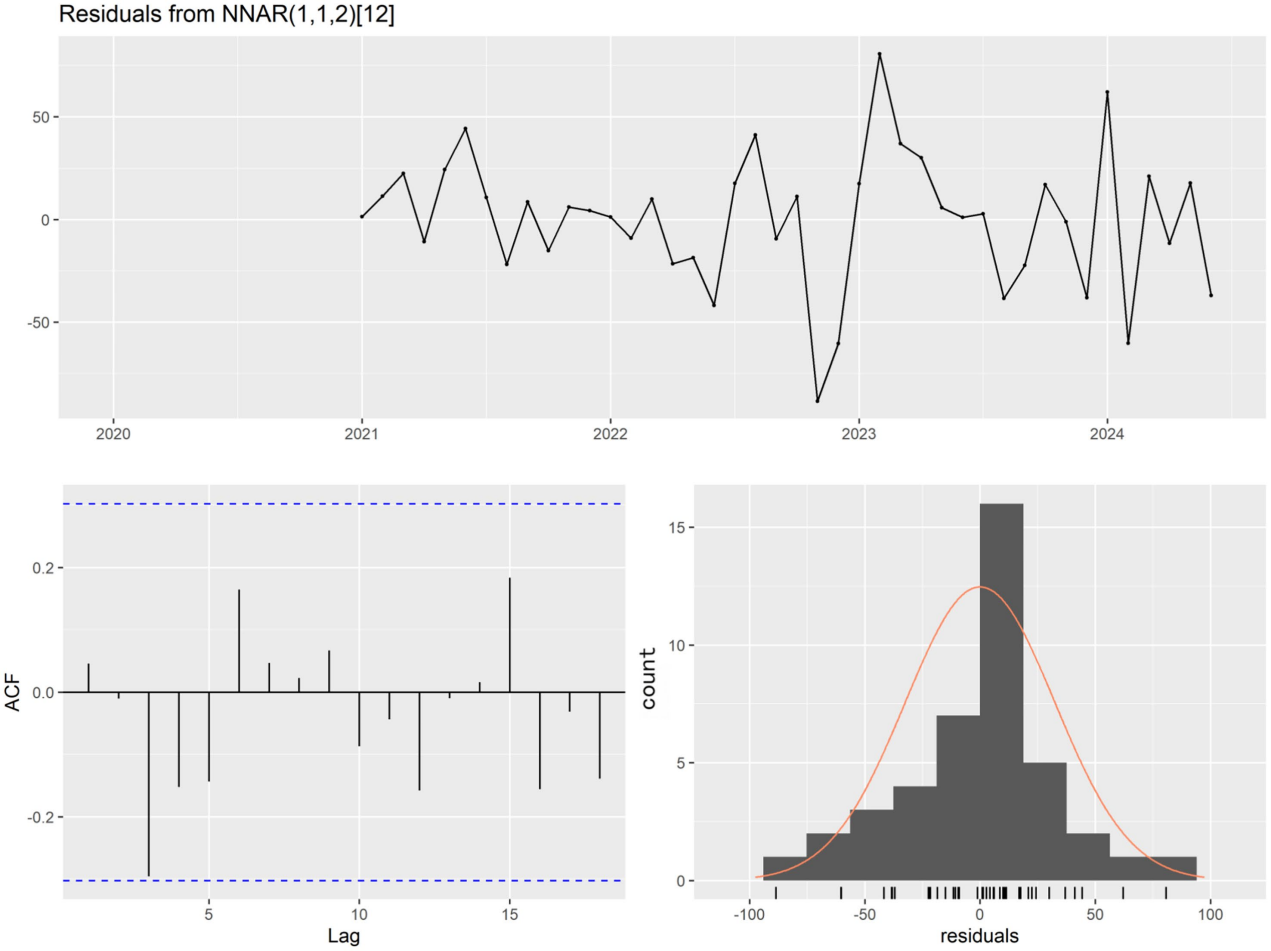
Residual plot, corresponding ACF plot, and histogram of NNAR (1,1,2)_12_.

For the seasonal component of this data, P was set to 1. The model was automatically generated by the γnnetar() function from the forecast package. The optimal forecasting model ultimately obtained was NNAR (1,1,2) (12). The Ljung–Box test conducted on this model yielded a *p*-value of 0.6468, indicating no autocorrelation in the residuals (Figure 6).

### 3.3 Performance of the hybrid model

**Figure 7:**
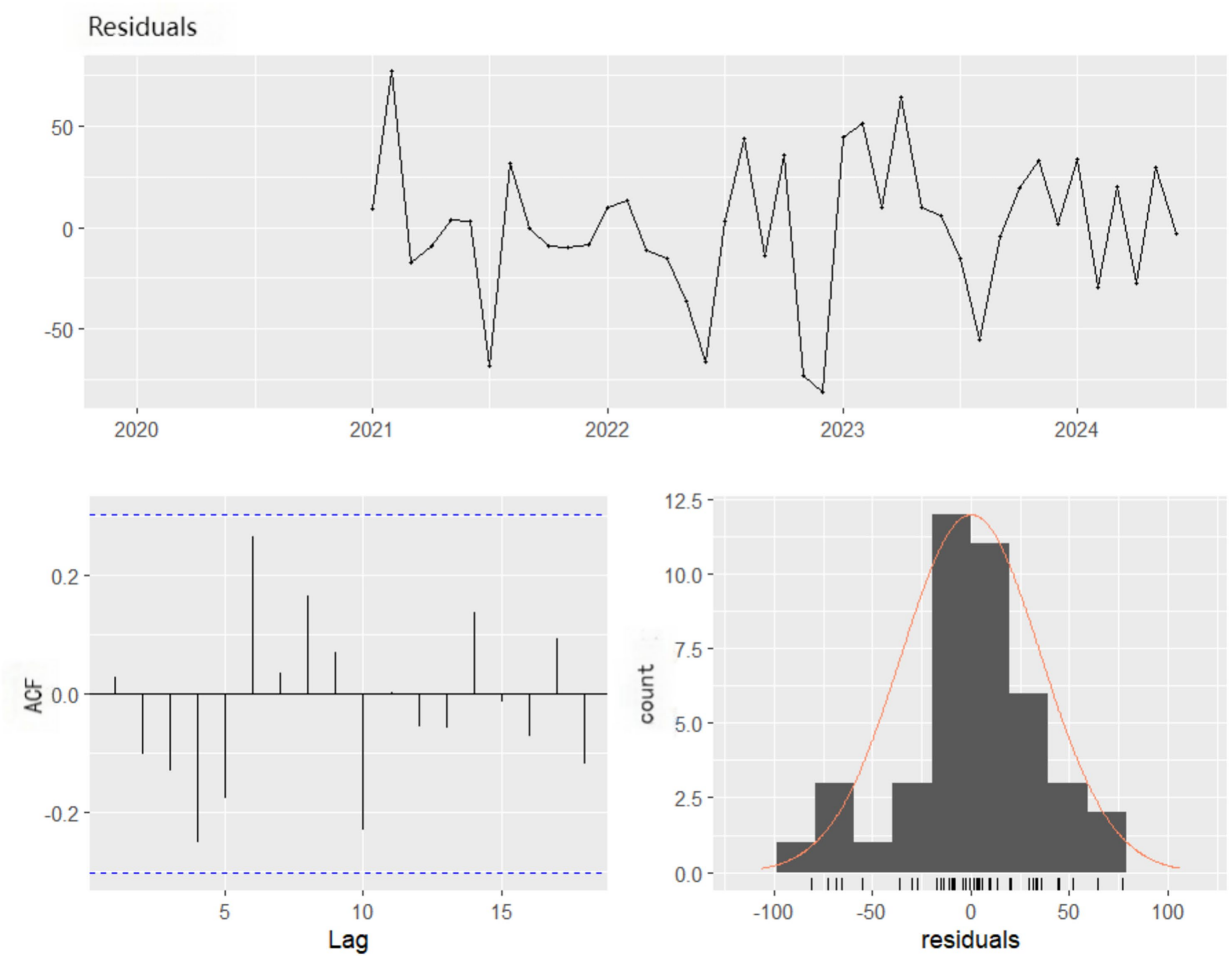
Residual plot, corresponding ACF plot, and histogram of hybrid.

The optimal SARIMA (0,0,1)(1,1,0) (12) and the optimal NNAR (1,1,2) (12) models were combined to construct the Hybrid model. The SARIMA residuals, which served as the input to the NNAR component, were confirmed to be stationary via the Augmented Dickey–Fuller test (*p* < 0.01). The NNAR model was trained solely on the lagged values of these residuals, without including lagged terms of the original case series, ensuring that only the remaining nonlinear fluctuations were modeled. The Ljung–Box test conducted on this model yielded a p-value of 0.2579, indicating no autocorrelation in the residuals (Figure 7).

### 3.4 Accuracy assessment between models

The performance of the models in terms of fitting and forecasting effectiveness is as follows. The NNAR (1,1,2) (12) model exhibited the lowest values for RMSE, MAE, and MAPE in the training set. In the testing set, the hybrid model showed the lowest MAE and MAPE values, while the NNAR model had the lowest RMSE value. All models performed well in the training set, with MAPE values all below 10%. A comprehensive comparison of the predictive performance of the three models on the testing set indicated that the time series fitted by the hybrid model was closest to the original data, followed by the NNAR model (Table 2).

**Table 2:** Comparison of the fit and prediction accuracy of three models.

Models	Training set			Testing set		
	RMSE	MAE	MAPE	RMSE	MAE	MAPE
SARIMA(0,0,1)(1,1,0) [12]	39.10	25.54	8.98	67.02	48.82	14.83
NNAR(1,1,2) [12]	32.10	24.12	8.21	45.52	43.83	13.66
Hybrid	35.08	26.35	9.05	46.26	38.39	11.77

### 3.5 Predicted values of different models

**Figure 8:**
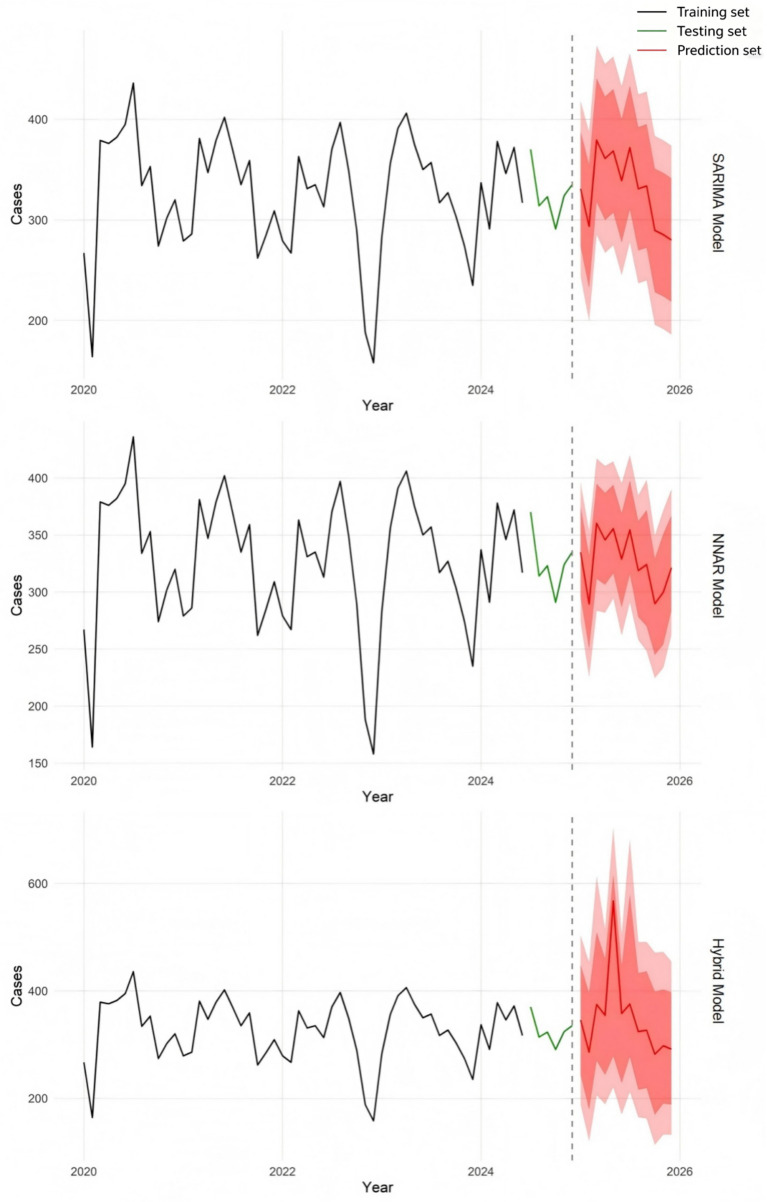
Forecasts from three models.

The number of PTB cases among older adults from January 2025 to December 2025 was forecasted using the SARIMA, NNAR, and SARIMA-NNAR models (Table 3). The predicted time series plots from the SARIMA, NNAR, and hybrid models indicate that peaks in the case counts of PTB among older adults are projected to occur in March and May 2025 (Figure 8).

**Table 3:** Comparison of model predictions from January to December 2025.

Time	SARIMA 95% CI	NNAR 95% CI	Hybrid 95% CI
January 2025	331(243,418)	335(274,396)	346(188,503)
February 2025	294 (200,387)	290 (225,353)	286 (121,453)
March 2025	379 (286,473)	360 (284,417)	375 (206,614)
April 2025	361 (267,455)	346 (282,410)	354 (189,515)
May 2025	369 (275,462)	356 (295,414)	567 (221,704)
June 2025	339 (245,433)	329 (262,395)	358 (170,504)
July 2025	372 (278,466)	354 (291,420)	376 (212,683)
August 2025	331 (237,425)	319 (258,385)	324 (165,491)
September 2025	334 (240,428)	324 (248,398)	327 (162,491)
October 2025	290 (196,383)	290 (225,350)	282 (114,471)
November 2025	285 (192,379)	300 (234,372)	298 (133,473)
December 2025	280 (186,374)	321 (263,390)	291 (132,455)

## 4 Discussion

This study found that the number of PTB cases among older adults in Chongqing exhibits distinct seasonal peaks, primarily concentrated in March and May each year. This pattern may be associated with climatic factors, crowd-gathering activities, and seasonal fluctuations in immune function (35–37). It is also largely consistent with the seasonal characteristics of TB cases reported in other regions domestically and internationally. For example, the peak of TB disease of TB in Turkey occurs in spring, specifically April (38); in Japan, the peak of TB disease is observed in spring and winter (39); while in Guangxi, China, the number of TB cases peaks in spring or summer (40).

Based on the monthly case counts of PTB among the population aged 65 and above in Chongqing from 2020 to 2024, this study constructed and compared the fitting and predictive performance of the SARIMA model, the NNAR model, and the SARIMA-NNAR hybrid model. The results indicate that all three models could adequately capture the seasonal fluctuation characteristics of PTB case counts among older adults. With respect to model comparison, the NNAR model achieved the lowest error values on the training set, reflecting its ability to capture nonlinear patterns in the series that a purely linear SARIMA model cannot fully represent (41). On the testing set, the hybrid model attained the lowest MAE and MAPE among the three models, whereas the NNAR model yielded a slightly lower RMSE. As a classic linear time series model, SARIMA demonstrated stable performance in capturing seasonal variations, but its adaptability to nonlinear fluctuations was weaker, resulting in the highest prediction error on the testing set. The comparatively larger increase in NNAR error metrics from the training to the testing phase is consistent with a recognized characteristic of flexible nonlinear models: they tend to fit historical data closely but may capture training-set-specific fluctuations that do not generalize well to new data (42). The SARIMA–NNAR hybrid model mitigates this tendency by confining the neural network to the SARIMA residuals, which have a mean near zero and reduced variance, thereby preserving nonlinear modeling capacity while achieving greater stability in average error magnitude and percentage error on new data. (24) Given its more balanced performance across evaluation metrics, the hybrid model was selected as the final model to forecast the number of PTB cases among older adults in Chongqing for 2025. The forecasting results indicated that the case counts in 2025 would continue to exhibit a similar seasonal pattern, suggesting that prevention and control efforts should strengthen monitoring and interventions prior to seasonal peak periods.

A notable observation is the hybrid model’s elevated forecast for May 2025 (567 cases), which exceeds both the historical May average (377) and the standalone SARIMA (369) and NNAR (356) forecasts. This anomaly does not stem from residual outliers (range: −113 to +101) but rather reflects the NNAR component’s propensity to amplify minor systematic patterns in the training residuals—a recognized limitation of neural network forecasting (43). The R documentation for nnetar similarly notes that overly complex networks may overfit and produce unstable out-of-sample predictions (44). Importantly, the 95% prediction interval for May 2025 (221–704) encompasses historically plausible values, underscoring the necessity of reporting intervals alongside point forecasts. This case illustrates that the hybrid model’s overall accuracy gains (lower MAE/MAPE) do not preclude occasional overfitting to localized residual structures. Future studies could apply residual regularization or ensemble averaging to mitigate such artifacts.

It is also important to consider the potential influence of the COVID-19 pandemic on the training data. During 2020–2022, lockdowns, healthcare disruptions, and population-wide public health measures in China may have temporarily altered PTB case detection and reporting among older adults (45). As the entire training set of this study falls within or shortly after the pandemic period, the temporal patterns captured by the models may partly reflect pandemic-related artifacts rather than purely endemic transmission dynamics. However, the absence of a sustained declining trend in the monthly case counts during 2020–2024 suggests that any pandemic-induced underreporting was likely transient and did not fundamentally distort the overall seasonal structure. Additionally, the monthly case counts for November and December 2022 (188 and 158, respectively) were notably lower than those of the same months in other years. These 2 months coincided with the period around the declaration of the end of the COVID-19 pandemic in China in December 2022. During this transitional phase, adjustments to prevention and control measures, together with heightened caution among older adults toward visiting healthcare facilities, may have led to delayed care-seeking and a temporary reduction in the number of patients presenting for TB diagnosis (46). The seasonal pattern returned to its typical levels in 2023, indicating that the low values in late 2022 represented a transient fluctuation in the surveillance data. Future studies with extended pre-pandemic baseline data would be valuable to disentangle pandemic effects from underlying disease patterns.

The clinical diagnosis of PTB in older adult populations presents persistent challenges, particularly given the frequent atypical presentations in this age group. Fever of unknown origin studies in China have identified tuberculosis as a leading infectious cause, underscoring the diagnostic difficulty that may contribute to underdetection of older adults PTB cases (47). In older patients, distinguishing PTB from lung cancer is particularly relevant, as expert CT interpretation plays a critical role in determining the probability of malignancy when pathological confirmation is unavailable (48). Large-scale molecular profiling initiatives such as the CLEVER study have been established to characterize the evolution and microenvironment of lung cancer in Chinese populations, further emphasizing the importance of accurate differential diagnosis in older adults patients with suspected PTB (49). Advances in non-invasive imaging have also expanded the diagnostic and monitoring toolkit; hyperpolarized gas magnetic resonance imaging, for example, enables visualization of regional lung ventilation without ionizing radiation and has been explored in preclinical lung models (50, 51). These clinical and technological developments suggest that future forecasting models may benefit from incorporating imaging-derived or molecular variables to complement case-count-based predictions.

This study demonstrates that the SARIMA-NNAR hybrid model, through complementary strengths, achieves higher prediction accuracy and stability. This conclusion is supported by evidence from multiple settings. A study conducted in Qinghai Province, China, combined the SARIMA model with the NNAR model to predict PTB disease and found that the hybrid model exhibited superiority in both the fitting and forecasting phases (52). In India, researchers compared SARIMA, NNAR, and their hybrid model, finding that the combined model performed better on the prediction set, with lower forecast errors than individual time series models (53). These studies collectively indicate that for TB epidemics driven by multiple factors such as social and environmental influences, a hybrid modeling strategy integrating linear and nonlinear methods is an effective approach to improving forecast reliability. The application of this strategy in the key older adult population in Chongqing in this study further validates its practical value.

It should be noted that the forecasting design employed in this study is based on a relatively short time series (54 months for training) and a six-month testing horizon. A single six-month testing period, while sufficient to assess short-term predictive accuracy, covers only half of a full seasonal cycle and may not fully capture the model’s performance across all seasons. Furthermore, period-specific factors-such as unusual weather patterns, healthcare policy adjustments, or local disease outbreaks-could influence the reported accuracy metrics (54). As such, the predicted values for 2025 represent a statistical extrapolation of recent temporal patterns under the assumption of a stable data-generating process, and should be interpreted as plausible scenarios rather than definitive projections. In practice, the upper bounds of the 95% prediction intervals reported in Table 3 can serve as a reference for resource stockpiling and surge capacity preparation ahead of anticipated seasonal peaks (55).

Based on the model’s forecasts for 2025, prevention and control resources can be allocated more efficiently and dynamically, thereby enhancing the timeliness and cost-effectiveness of interventions. The prediction model established in this study can provide a quantitative reference for the prevention and control of PTB among older adults in Chongqing. These forecasts may inform the timing of enhanced surveillance and preventive activities. Specifically, based on the projected peaks in March and May 2025, intensified screening and health education campaigns could be prioritized in the month preceding the anticipated seasonal increases, when resource mobilization would be most impactful. However, such applications should consider the inherent uncertainty of statistical forecasts and be integrated with real-time epidemiological intelligence.

### 4.1 Limitations

Despite the hybrid model demonstrating relatively good predictive performance, this study still has several limitations. First, the original data were derived from cases reported and registered by primary healthcare institutions, which may involve misreporting and underreporting. Moreover, notified case counts reflect the product of the number of true disease cases and the case detection rate, which may vary over time with changes in screening intensity, diagnostic capacity, or healthcare-seeking behavior. Consequently, temporal patterns in the reported data may partly reflect variations in detection rather than genuine changes in transmission, and this distinction should be considered when interpreting the model forecasts. Second, the relatively short time span of the data, together with the six-month testing period covering only a partial seasonal cycle, may limit the ability to identify long-term trends and robustly assess seasonal forecasting performance. Third, the model is based solely on case numbers for prediction and does not incorporate external influencing factors such as meteorological conditions, socioeconomic factors, healthcare policy changes, the introduction of new diagnostic tools, or the distribution of healthcare resources, which limits the model’s explanatory and early-warning capabilities. Furthermore, because the models predict absolute monthly case counts rather than population-adjusted incidence rates, they do not account for changes in the size or proportion of older adult population over time. In the context of Chongqing’s aging population-which reached 6.02 million in 2024, accounting for 18.87% of the total population-stable predicted case counts may, in fact, reflect a declining incidence rate, an important distinction that the current univariate framework cannot capture. Finally, it should be noted that this study did not employ more complex deep learning architectures such as LSTM-based hybrid models. Given the relatively short length of the time series (54 training observations), the SARIMA-NNAR framework was preferred as it offers a parsimonious yet flexible approach with established reproducibility. Future studies with longer time series or multi-regional data may explore SARIMA-LSTM or comparable deep learning hybrids and compare their predictive performance with the SARIMA-NNAR approach.

## 5 Conclusion

This study employed a SARIMA–NNAR hybrid model to forecast the monthly number of PTB cases among older adults in Chongqing for 2025. On the testing set, the hybrid model achieved the lowest MAE and MAPE among the three models, while the NNAR model yielded a slightly lower RMSE. Overall, the hybrid model demonstrated a more balanced performance across evaluation metrics. These results indicated that integrating linear and nonlinear approaches could enhance forecasting accuracy. The predicted seasonal trends suggest possible case peaks in March and May 2025, which may provide a reference for the timing of intensified prevention and control efforts; however, given the inherent uncertainty of statistical extrapolation, the specific point estimates should be interpreted with caution.

## Data Availability

The data analyzed in this study is subject to the following licenses/restrictions: the data comes from the China Tuberculosis Information Management System (TBIMS) and constitutes legally reported infectious disease data. This dataset contains sensitive patient information and is governed by national public health data management regulations, and is not publicly available. Researchers must obtain approval from the relevant health administrative departments before accessing it. The data used in this study has undergone ethical review by the Chongqing Tuberculosis Prevention and Treatment Institute (Approval No.: 2025008), and its use is limited to the purposes of this study. To access the data, interested parties can contact the authors and explain the situation to the Chongqing Tuberculosis Prevention and Treatment Institute to obtain it. Requests to access these datasets should be directed to Bojie Gao, 18982276448@163.com.

## References

[1] World Health Organization (2025). Global Tuberculosis Report 2025. World Health Organization. Available online at: https://www.who.int/teams/global-programme-on-tuberculosis-and-lung-health/tb-reports/global-tuberculosis-report-2025 (Accessed December 8, 2025).

[2] Jiang H Chen X Lv J Dai B Liu Q Ding X . Prospective cohort study on tuberculosis incidence and risk factors in the elderly population of eastern China. Heliyon. (2024) 10:e24507. doi: 10.1016/j.heliyon.2024.e24507, 38314308 PMC10837496

[3] Zhang CY Zhao F Xia YY Yu YL Shen X Lu W . Prevalence and risk factors of active pulmonary tuberculosis among elderly people in China: Apopulation based cross-sectional study. Infect Dis Poverty. (2019) 8:7. doi: 10.1186/s40249-019-0515-y, 30654836 PMC6337869

[4] Chen Q Peng L Xiong G Peng Y Luo D Zou L . Recurrence is a noticeable cause of rifampicin-resistant *mycobacterium tuberculosis* in the elderly population in Jiangxi, China. Front Public Health. (2019) 7:182. doi: 10.3389/fpubh.2019.00182, 31380332 PMC6659409

[5] Chongqing Municipal People’s Government. Statistical Communiqué of Chongqing on the 2024 National Economic and Social Development. Chongqing Municipal Bureau of Statistics. Available online at: https://www.cq.gov.cn/zwgk/zfxxgkzl/fdzdgknr/tjxx/tjgb/202503/t20250326_14569744.html (Accessed December 8, 2025).

[6] Yu Y Hu XJ Fang XM Wu J. Epidemiology and treatment outcomes of pulmonary tuberculosis in dazu district, Chongqing, China, 2005-2024: surveillance study. JMIR Public Health Surveill. (2025) 11:e78564. doi: 10.2196/78564, 41328038 PMC12669922

[7] Wu B Yu Y Xie W Liu Y Zhang Y Hu D . Epidemiology of tuberculosis in Chongqing, China: a secular trend from 1992 to 2015. Sci Rep. (2017) 7:7832. doi: 10.1038/s41598-017-07959-2, 28798367 PMC5552739

[8] Xian S Yu Y Chen J Wu B Wu C Fan J . Spatiotemporal distribution and time-series analysis of rifampicin-resistant tuberculosis in Chongqing municipality, China. Public Health. (2025) 242:165–71. doi: 10.1016/j.puhe.2025.03.002 40090059

[9] Lv H Chen H Zhang X Li X Liu L Dang C . Analyzing factors affecting tuberculosis incidence in various mainland Chinese economic regions and predicting trends: a comprehensive regression study. BMC Public Health. (2025) 25:3282. doi: 10.1186/s12889-025-24575-2, 41034927 PMC12487523

[10] Ab Rashid MA Ahmad Zaki R Wan Mahiyuddin WR Yahya A. Forecasting new tuberculosis cases in Malaysia: a time-series study using the autoregressive integrated moving average (ARIMA) model. Cureus. (2023) 15:e44676. doi: 10.7759/cureus.44676, 37809275 PMC10552684

[11] Selmane S L’Hadj M. Forecasting and prediction of scorpion sting cases in Biskra province, Algeria, using a seasonal autoregressive integrated moving average model. Epidemiol Health. (2016) 38:e2016044. doi: 10.4178/epih.e2016044, 27866407 PMC5177803

[12] Wang KW Deng C Li JP Zhang YY Li XY Wu MC. Hybrid methodology for tuberculosis incidence time-series forecasting based on ARIMA and a NAR neural network. Epidemiol Infect. (2017) 145:1118–29. doi: 10.1017/S0950268816003216, 28115032 PMC9507834

[13] Tomov L Chervenkov L Miteva DG Batselova H Velikova T. Applications of time series analysis in epidemiology: literature review and our experience during COVID-19 pandemic. World J Clin Cases. (2023) 11:6974–83. doi: 10.12998/wjcc.v11.i29.6974, 37946767 PMC10631421

[14] Swaraj A Verma K Kaur A Singh G Kumar A Melo de Sales L. Implementation of stacking based ARIMA model for prediction of Covid-19 cases in India. J Biomed Inform. (2021) 121:103887. doi: 10.1016/j.jbi.2021.103887, 34407487 PMC8364768

[15] Gan R Chen N Huang D. Comparisons of forecasting for hepatitis in Guangxi province, China by using three neural networks models. PeerJ. (2016) 4:e2684. doi: 10.7717/peerj.2684, 27843718 PMC5103820

[16] Zhang Z. A gentle introduction to artificial neural networks. Ann Transl Med. (2016) 4:370. doi: 10.21037/atm.2016.06.20, 27826573 PMC5075856

[17] Bandara WMS Mel WARD. Forecasting nonlinear time series with ARIMA, ANN, and hybrid models: a case study on inflation rate in Sri Lanka. Indon J Stat Appl. (2025) 9:145–56. doi: 10.29244/ijsa.v9i1p145-156

[18] Limbu Sanwa R Khadka R Chi YN. Forecasting global monthly cotton prices: the superiority of NNAR models over traditional models. Front Artif Intell. (2025) 8:1628744. doi: 10.3389/frai.2025.1628744, 41425054 PMC12711705

[19] Alsheheri G. Comparative analysis of ARIMA and NNAR models for time series forecasting. J Appl Math Phys. (2025) 13:267–80. doi: 10.4236/jamp.2025.131012

[20] Olney B Mahmud S Karam R. Efficient nonlinear autoregressive neural network architecture for real-time biomedical applications. In: 2022 IEEE 4th International Conference on Artificial Intelligence Circuits and Systems (AICAS). Incheon, Republic of Korea: Institute of Electrical and Electronics Engineers (IEEE). (2022): 411–414.

[21] Yu G Feng H Feng S Zhao J Xu J. Forecasting hand-foot-and-mouth disease cases using wavelet-based SARIMA–NNAR hybrid model. PLoS One. (2021) 16:e0246673. doi: 10.1371/journal.pone.0246673, 33544752 PMC7864434

[22] Liu M Liu Y Liu J. Machine learning for infectious disease risk prediction: a survey. ACM Comput Surv. (2025) 57:212:1-212:39. doi: 10.1145/3719663

[23] Azeez A Obaromi D Odeyemi A Ndege J Muntabayi R. Seasonality and trend forecasting of tuberculosis prevalence data in eastern cape, South Africa, using a hybrid model. Int J Environ Res Public Health. (2016) 13:757. doi: 10.3390/ijerph13080757, 27472353 PMC4997443

[24] Akermi SE L’Hadj M Selmane S. Epidemiology and time series analysis of human brucellosis in Tebessa province, Algeria, from 2000 to 2020. J Res Health Sci. (2022) 22:e00544. doi: 10.34172/jrhs.2022.79, 36511254 PMC9315461

[25] Qureshi H. Artificial neural network simulation modeling of heat transfer effects on a magnetohydrodynamic Casson liquid film flow over a stretching surface. Multiscale and Multidiscip Model Exp and Des. (2026) 9:49. doi: 10.1007/s41939-025-01110-9

[26] Qureshi H. AI-driven analysis of buoyancy-convective flow of ternary-hybrid nanofluid in a porous medium over stretching cylinder. Nonlinear Dyn. (2025) 113:28907–24. doi: 10.1007/s11071-025-11620-3

[27] Qureshi H Alakhras AI. Ai-driven multilayer modeling of tetra-hybrid Casson nanofluid flow with thermal radiation: implications for solar energy and energy conversion. Results Phys. (2025) 79:108505. doi: 10.1016/j.rinp.2025.108505

[28] Qureshi H Zubair M Altmeyer SA. Machine learning investigation of ternary-hybrid radiative Nanofluid over stretching and porous sheet. Nanomaterials. (2025) 15:1525. doi: 10.3390/nano15191525, 41090870 PMC12526173

[29] Qureshi H Altmeyer S Zubair M. Machine learning investigation of marangoni convection in hybrid nanofluids with Darcy-Forchheimer. Sci Rep. (2025) 15:39657. doi: 10.1038/s41598-025-23362-8, 41224937 PMC12612216

[30] Chongqing Municipal People’s Government Website (2024). Chongqing Statistical Yearbook. Chongqing Municipal Bureau of Statistics. Available online at: https://tjj.cq.gov.cn/zwgk_233/tjnj/ (Accessed December 12, 2025).

[31] Hyndman R. J. Athanasopoulos G., Forecasting: Principles and Practice, Heathmont: Otexts, 2nd (2018). Available online at: https://otexts.com/fpp2/ (Accessed December 12, 2025).

[32] Mao Q Zhang K Yan W Cheng C. Forecasting the incidence of tuberculosis in China using the seasonal auto-regressive integrated moving average (SARIMA) model. J Infect Public Health. (2018) 11:707–12. doi: 10.1016/j.jiph.2018.04.009, 29730253 PMC7102794

[33] Jian Y Zhu D Zhou D Li N Du H Dong X . ARIMA model for predicting chronic kidney disease and estimating its economic burden in China. BMC Public Health. (2022) 22:2456. doi: 10.1186/s12889-022-14959-z, 36585665 PMC9801144

[34] Zenia S L'Hadj M Selmane S. A hybrid approach based on seasonal autoregressive integrated moving average and neural network autoregressive models to predict scorpion sting incidence in El Oued Province, Algeria, from 2005 to 2020. J Res Health Sci. (2023) 23:e00586. doi: 10.34172/jrhs.2023.121, 38315901 PMC10660509

[35] Wang X Guo J Shi X Cui L. Analysis of the epidemiological characteristics of pulmonary tuberculosis in Shijiazhuang, China 2010–2023. Front Public Health. (2025) 13:1621695. doi: 10.3389/fpubh.2025.1621695, 40678650 PMC12267174

[36] Zhang Y Ye J Hou S Lu X Yang C Pi Q . Spatial-temporal analysis of pulmonary tuberculosis in Hubei province, China, 2011-2021. PLoS One. (2023) 18:e0281479. doi: 10.1371/journal.pone.0281479, 36749779 PMC9904469

[37] Kong D Xia Y Wang X Zhang Y Zhong J Zhang T . Prevalence trends, population characteristics and treatment outcomes of tuberculosis combined with diabetes in Southwest China: a register-based retrospective study. Front Public Health. (2024) 12:1445857. doi: 10.3389/fpubh.2024.1445857, 39635223 PMC11616032

[38] Taylan M Dogru S Sezgi C Yilmaz S. Epidemiological trends and seasonal dynamics of tuberculosis in southeastern Turkey. Niger J Clin Pract. (2023) 26:928–33. doi: 10.4103/njcp.njcp_629_22, 37635576

[39] Manabe T Takasaki J Kudo K. Seasonality of newly notified pulmonary tuberculosis in Japan, 2007-2015. BMC Infect Dis. (2019) 19:497. doi: 10.1186/s12879-019-3957-8, 31170932 PMC6555020

[40] Cui Z Lin D Chongsuvivatwong V Zhao J Lin M Ou J. Spatiotemporal patterns and ecological factors of tuberculosis notification: a spatial panel data analysis in Guangxi, China. PLoS One. (2019) 14:e0212051. doi: 10.1371/journal.pone.0212051, 31048894 PMC6497253

[41] Almarashi AM Daniyal M Jamal F. A novel comparative study of NNAR approach with linear stochastic time series models in predicting tennis player's performance. BMC Sports Sci Med Rehabil. (2024) 16:28. doi: 10.1186/s13102-024-00815-7, 38273407 PMC10809504

[42] Peng Y Nagata MH. An empirical overview of nonlinearity and overfitting in machine learning using COVID-19 data. Chaos Solitons Fractals. (2020) 139:110055. doi: 10.1016/j.chaos.2020.110055, 32834608 PMC7324351

[43] Taskaya-Temizel T Casey MC. A comparative study of autoregressive neural network hybrids. Neural Netw. (2005) 18:781–9. doi: 10.1016/j.neunet.2005.06.003, 16085389

[44] Hyndman R. J. Caceres G. (n.d.). Forecast.Nnetar: forecasting using neural network models. In Forecast: Forecasting Functions for time series and linear Models (Version 8.21) [R Package]. Available online at: https://api.rdocumentation.org/packages/forecast/versions/8.21/topics/forecast.nnetar (Accessed April 21, 2026).

[45] Wang X He W Lei J Liu G Huang F Zhao Y. Impact of COVID-19 pandemic on pre-treatment delays, detection, and clinical characteristics of tuberculosis patients in Ningxia Hui autonomous region, China. Front Public Health. (2021) 9:644536. doi: 10.3389/fpubh.2021.644536, 34095053 PMC8175850

[46] Zhang G Yu Y Zhang W Shang J Chen S Pang X . Influence of COVID-19 for delaying the diagnosis and treatment of pulmonary tuberculosis-Tianjin, China. Front Public Health. (2022) 10:937844. doi: 10.3389/fpubh.2022.937844, 36530737 PMC9755169

[47] Kang S Zheng R. Distribution of the causes of fever of unknown origin in China, 2013-2022. J Transl Int Med. (2024) 12:299–307. doi: 10.2478/jtim-2024-0008, 39081273 PMC11284625

[48] Nakamura D Honda K Yamazaki T Hayashi H Tsutsui S Fukushima A . Stereotactic body radiation therapy for clinically diagnosed early-stage non-small cell lung cancer: importance of accurate CT interpretation by experts. Precis Radiat Oncol. (2024) 8:30–6. doi: 10.1002/pro6.1220, 40336564 PMC11935021

[49] Wang W He Y Yuan X Weng L Bai J Liu X . Protocol for Chinese lung cancer evolution and microenvironment tracking under therapy study. J Transl Int Med. (2025) 13:599–609. doi: 10.1515/jtim-2025-0047, 41438463 PMC12721363

[50] Chowdhury MRH Oladun C Ariyasingha NM Samoilenko A Bawardi T Burueva DB . Rapid lung ventilation MRI using parahydrogen-induced polarization of propane gas. Analyst. (2024) 149:5832–42. doi: 10.1039/d4an01029a, 39530397 PMC11563306

[51] Ariyasingha NM Oladun C Samoilenko A Chowdhury MRH Nantogma S Shi Z . Parahydrogen-hyperpolarized propane-d6 gas contrast agent: T1 relaxation dynamics and pilot millimeter-scale ventilation MRI. J Phys Chem A. (2025) 129:4275–87. doi: 10.1021/acs.jpca.4c08800, 40311080 PMC12633766

[52] Wang Y Xu C Li Y Wu W Gui L Ren J . An advanced data-driven hybrid model of SARIMA-NNNAR for tuberculosis incidence time series forecasting in Qinghai Province, China. Infect Drug Resist. (2020) 13:867–80. doi: 10.2147/IDR.S232854, 32273731 PMC7102880

[53] Yadav BK Srivastava SK Arasu PT Singh P. Time series modeling of tuberculosis cases in India from 2017 to 2022 based on the SARIMA-NNAR hybrid model. Can J Infect Dis Med Microbiol. (2023) 2023:5934552. doi: 10.1155/2023/5934552, 38144388 PMC10748728

[54] Castro LA Shelley CD Osthus D Michaud I Mitchell J Manore CA . How New Mexico leveraged a COVID-19 case forecasting model to preemptively address the health care needs of the state: quantitative analysis. JMIR Public Health Surveill. (2021) 7:e27888. doi: 10.2196/27888, 34003763 PMC8191729

[55] Johnson MR Naik H Chan WS Greiner J Michaleski M Liu D . Forecasting ward-level bed requirements to aid pandemic resource planning: lessons learned and future directions. Health Care Manag Sci. (2023) 26:477–500. doi: 10.1007/s10729-023-09639-2, 37199873 PMC10191824

