# Supplementary figures and images for: Prediction of pulmonary tuberculosis case trends among older adults in Chongqing based on time series models
Source: Front Public Health. 2026 May 18;14:1839570. doi: 10.3389/fpubh.2026.1839570 (PMC13223132; doi:10.3389/fpubh.2026.1839570)

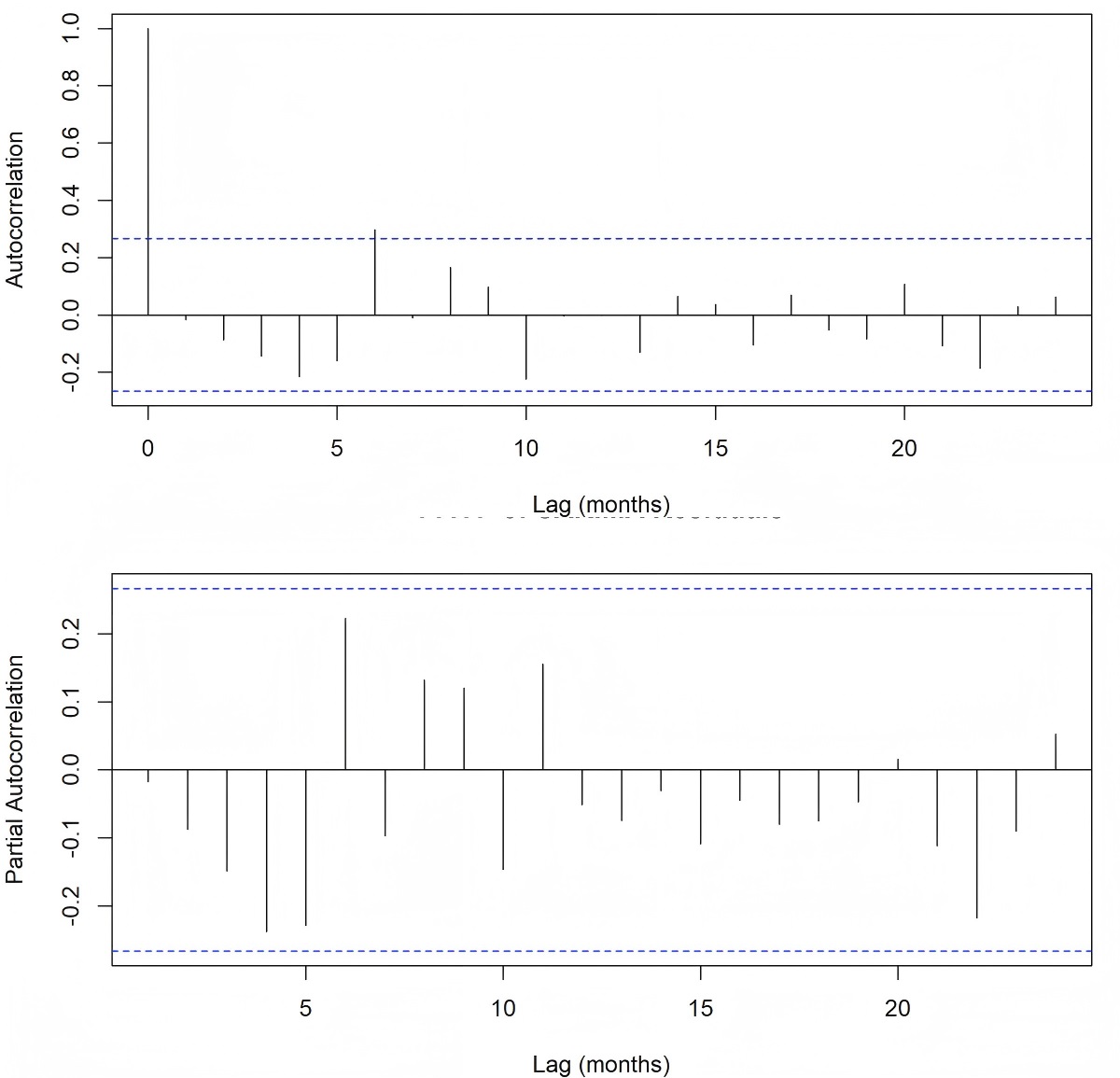

Supplement: Supplementary file 1 [file Image_1.jpeg]
